# English use anxiety, motivation, self-efficacy, and their predictive effects on Chinese top university students’ English achievements

**DOI:** 10.3389/fpsyg.2022.953600

**Published:** 2022-10-19

**Authors:** Xia Wu, Huameng Yang, Junxia Liu, Ziyu Liu

**Affiliations:** ^1^Department of Foreign Languages and Literatures, Tsinghua University, Beijing, China; ^2^Academic Affairs Office, Tsinghua University, Beijing, China

**Keywords:** English use anxiety, motivation, self-efficacy, top-notch program, EFL

## Abstract

The present study examined English use anxiety, motivation, self-efficacy, use of English, and their predictive effects on top university students’ English achievements. Two hundred and twenty-three students of the Top-Notch Students of Basic Disciplines Training Program in a top Chinese university answered a battery of questionnaires, which consisted of the 8-item English Use Anxiety Questionnaire, the 5-item Motivational Self-Talk Questionnaire, the 3-item Self-Efficacy Questionnaire, the 19-item Language Learning Orientations Questionnaire, and a Background Information Questionnaire. Analyses of the data revealed the following major findings: (1) the participants had a low level of extrinsic motivation-introjected regulation, a low-to-medium level of English use anxiety, extrinsic motivation-external regulation, intrinsic motivation-knowledge, and a medium-to-high level of motivational self-talk, self-efficacy, extrinsic motivation-identified regulation, intrinsic motivation-accomplishment, and intrinsic motivation-stimulation, (2) use of English anxiety (UAE) and language learning orientation were generally significantly negatively correlated with each other, and significantly correlated with other measured variables, (3) UAE and intrinsic motivation-knowledge significantly predicted the participants English achievements, measured both by standardized test scores and self-rated overall English proficiency, and (4) use of English and self-efficacy mediated the effects of English use anxiety and language learning orientations on the participants’ English achievements. These findings further pinpoint the importance of anxiety and motivation in second/foreign language learning.

## Introduction

Many studies have proven that besides being influenced by teachers and teaching materials, second language (L2) learners’ achievement can be affected by their own individual factors such as gender, age, anxiety, proficiency in the target language, self-confidence, motivation, and self-efficacy (Deci and Ryan, 1985; Bandura, 1997). It is generally found that contact with the target language, self-confidence, self-efficacy, and motivation significantly positively correlate and predict L2 learning outcomes, while anxiety, limited access to the target language, and demotivation significantly negatively correlate with the latter (Dikmen et al., 2016; Gong et al., 2020; Dong et al., 2022; Liu and Zhang, 2022). For example, Gong et al. (2020) and Liu and Zhang (2022) found that contact with native speakers increased participants’ interest in and motivation to learn the target language, which led to enhanced proficiency in the language. Dong et al. (2022) found that learners with lower anxiety and greater motivation usually performed better in the target language than those with greater anxiety and lower motivation. This might be because motivation offers second language learners the stimulus to start learning and encourages them to keep learning without feeling reluctant, and eventually helps them to meet their goals (Dörnyei, 2005). Also, learners with high self-efficacy are more confident about getting a good learning result, and they are more active in searching for and using efficient learning strategies to improve their language proficiency (Wong, 2005).

As reviewed in more detail below, contact with the target language, foreign language anxiety, language learning motivation, and self-efficacy are all important factors in L2 learning and acquisition. However, they have rarely been examined in relation to L2 learning outcomes simultaneously in the same context. Moreover, most research in second language acquisition focuses on ordinary students, with a few studies targeting good/successful learners, little research can be found on top students of top universities. In order to prepare leading scholars in basic disciplines, China’s Ministry of Education has been investing in top undergraduate students in top universities by providing them with (much) more learning and research resources during the recent decade. Nevertheless, little research has been done on these students to examine how they learn a foreign language. This is the aim of the current study, which sought to examine the profiles of English use anxiety, motivation, and self-efficacy of top students in Chinese top universities and how they affected the students’ learning of English, the primary foreign language in China, hoping to shed light on general English teaching and learning in higher education in China and elsewhere of the world.

## Literature review

### Foreign language anxiety

Studies about anxiety of foreign language learners can date back to the 1970s, but it was not identified as one separate anxiety type until mid-1980s (Horwitz, 2010), when scholars began to realize that such anxious feelings were provoked only by the experience of using and learning a second language. Bailey (1983) was among the first scholars who studied language learning anxiety from the perspective of learners. Two years later, in the study of attitudes and motivation in language learning, Gardner (1985) pointed out that there might be a specific type of anxiety generated in the process of second language acquisition. According to Horwitz et al. (1986), foreign language classroom anxiety (FLCA), which is also referred to as foreign language anxiety or language anxiety sometimes, is “a distinct complex of self-perceptions, beliefs, feelings, and behaviors related to classroom language learning arising from the uniqueness of the language learning process” (p. 128). In some other studies, this universal and unavoidable language anxiety was expanded to language experience outside the classroom, being described as the tension related to the usage of a second language that will “interfere with the acquisition, retention and production of the new language” (MacIntyre and Gardner, 1991, p. 86) or “the worry and usually negative emotional reaction aroused when learning or using an L2” (MacIntyre, 2007, p. 565).

Foreign language anxiety can affect the second language learning process in various ways and may interfere all three stages of language input, processing, and output, being especially problematic during the two latter stages (MacIntyre and Gardner, 1994). Horwitz et al. (1986) pointed out that such anxiety covered three dimensions: communication apprehension (being afraid of interacting with others), test anxiety (being afraid of failure), and fear of negative evaluation (being afraid of receiving negative comments from the others), and anxious learners may fail to understand the course contents, feel worried, try to avoid taking part in course-related activities, or have difficulty in presenting themselves orally. In the study of 92 English-speaking college students in French courses, Gardner and Mac Intyre (1993) discovered a significant correlation between the students’ anxiety and their performance in taking tests. They noticed that learners who were more anxious would underrate their test results and language ability. The anxious feeling of learning a second language may also interfere with language proficiency in general (Dewaele and Ip, 2013; Yim, 2014), hold back learners’ willingness of L2 production (MacIntyre et al., 1998), as well as performance in reading (Saito et al., 1999) and listening to a foreign language (Elkhafaifi, 2005). Similar problem may also occur when the learners feel anxious about memorizing what has been taught, for instance, vocabulary or grammar, which leads to the inability of producing complicated sentences or language structures (Onwuegbuzie et al., 1999).

To quantify L2 learners’ anxiety, Gardner (1985) included a French Class Anxiety Scale in his Attitudes and Motivation Test Battery, which was later adapted and applied to foreign language learners of different levels in various countries, including secondary school students in Ireland (Muircheartaigh and Hickey, 2008), college students in Myanmar (Khaing, 2021), and vocational school students in Turkey (Özer, 2019). Horwitz’s (Horwitz et al., 1986) team, on the other hand, developed the Foreign Language Classroom Anxiety Scale, which had 33 items and was then applied in studies of non-western languages, including Chinese (Sun and Teng, 2021), Japanese (Aida, 1994), Korean (Jee, 2016), and so on. Recent studies have indicated more focus on the idea of positive psychology, therefore leading to the development of Foreign Language Enjoyment questionnaire (Dewaele and Mac Intyre, 2014; Liu and Hong, 2021; Dong et al., 2022) as a new perspective of interpreting FLCA. For example, Liu and Hong (2021) collected quantitative and qualitative data from 709 primary and secondary school students in China. They found that the students tended to be more anxious and less joyful in the English class as their grade levels increased, and that the students reacted differently when feeling anxious or joyful in class. They suffered when feeling/becoming anxious, which negatively affected their learning of English, yet they often became more attentive and active in class and studied English harder. The researchers thus concluded that foreign language anxiety may negatively affect L2 learning in various ways and can take place regardless of learners’ target language, native language, language proficiency, and age.

Even though positive psychology is catching researchers’ increasing attention, anxiety is still an important factor affecting L2 learning and a topic of research in second language acquisition. And it is interesting to know whether and to what degree top students in Chinese top universities feel anxious in learning and using English.

### Language learning motivation

The relationship between learning attitudes and second language achievement has always been what researchers are interested about (Busato et al., 2000; Jiang et al., 2018; Saito et al., 2018). Gardner (1985) examined data collected from 33 studies of French learners in Canada and compared nine different criteria of language achievements with five measures of attitudes. His discoveries indicated that some categories of attitude are more closely related to gaining achievement of different types, and learning motivation is among the most influential factors. Studies have proven that learners with stronger motivation tend to set up higher goals, which then lead to better academic performance (Lou and Noels, 2017), and without the motivation of learning a second language, individuals cannot realize the goal of learning even if they are equipped with the best learning facilities (Dörnyei, 2005). Different from other subjects, language learning involves the learner’s understanding and attitudes toward the social and cultural features behind the language; therefore, the motivation of learning a second language cannot be fully generalized as the motivation of learning. Gardner (1985) believed that if a language learner is considered motivated, the person needs to at least be equipped with four things: “a goal, effortful behaviors, a desire to attain the goal and favorable attitudes toward the activity in question” (p. 50). Among these four aspects raised by Gardner, the latter three usually differ from individual to individual, and thus become the important elements to measure one’s learning motivation.

It is not easy to relate the complicated construct of motivation to actual behaviors and build up a certain single model, let alone the specific motivation of learning a second language (Kálmán and Eugenio, 2015). According to Dörnyei (2005), the development of studies about second language motivation started from a social psychological perspective. The researchers on the Canadian ethnolinguistic communities (English and French) conducted some of the most representative social psychological studies of L2 motivation, and the leading roles, Gardner and Lambert, have discovered the significant influence of L2 learning motivation on improving language proficiency and eventually strengthening interactions between cultures (Gardner and Lambert, 1972). Later, researcher turned to a cognitive-situated perspective, and more studies began to put emphasis on the learning experiences in a language classroom (Dörnyei, 2005). With more attention to the specific context of L2 learning, the self-determination theory analyzes L2 learner’s motivation from both intrinsic and extrinsic perspectives (Deci and Ryan, 1985). The intrinsic motivation (IM) indicates the willingness to conduct an activity because it brings the feeling of satisfaction, and in L2 learning, it is the “innate needs for competence and self-determination” (Noels et al., 2000, p. 61). IM can be divided into three types: IM – Knowledge describes the desire for new information, IM – Accomplishment aims at the realization of a goal, and IM-Stimulation is for the exciting feeling of participating throughout an assignment (Noels et al., 2000). The extrinsic motivation (EM), on the other hand, brings learners the energy to conduct the learning actions with an instrumental purpose, and based on the level of influence on the learners’ self-determination, EM can be divided into external regulation, introjected regulation, and identified regulation (Noels et al., 2000). In the research, Deci and Ryan (1985) proposed a continuum of different forms of extrinsic motivation, with amotivation and intrinsic motivation on the two ends (Kálmán and Eugenio, 2015), and they discovered that learners who are more motivated from the inside and receive more support from the surrounding social environment can be more self-determined in the language learning process (Deci and Ryan, 1985). Noels et al. (2000) further extended the idea and introduced a Language Learning Orientations Scale (LLOS-IEA) with seven correlated subscales, and the results suggest that intrinsic motivation and extrinsic motivation may appear in separate continuums but the general findings are consistent with the previous research. These definitions of extrinsic and intrinsic motivation have been gradually modified, specifically in the EFL context, for learners nowadays are largely motivated by the abundant international opportunities with the phenomenon of Global English (Lamb, 2003; Lanvers, 2017), and the increasing intercultural influence of learners’ attitudes toward language learning should also be considered during evaluations. Dong et al. (2022) explored the relations among foreign language (FL) classroom anxiety, enjoyment, expectancy-value motivation, and their predictive effects on Chinese high school students’ self-rated FL proficiency. Two hundred and eighty senior high school Chinese English as FL learners answered a battery of questionnaires. The study showed that the students generally had a medium to a high level of FL classroom emotions and English learning motivation, and that motivation and anxiety jointly significantly predicted the students’ self-rated FL proficiency.

Despite the plethora of research on L2 motivation, more continuous research is needed on students with various backgrounds, since motivation is dynamic and is continuously shaped by the learning environment (Gong et al., 2020).

### Self-efficacy

The concept of self-efficacy was introduced by Bandura (1986) as a part of his social cognitive theory, and it refers to “people’s judgments of their capabilities to organize and execute courses of action required to attain designated types of performances” (p. 391). Self-efficacy can help the individual to decide which task to take, how much time and energy to spend on the task and how determined when encountering obstacles (Wang et al., 2018).

Bandura (1997) mentioned that four different sources can help to raise one’s self-efficacy: previous successful experience of participating in the same or similar activities, seeing others dealing with the same task easily, encouragement or recognition from others and the positive emotional status of one’s own. In the context of learning a second language, studies have shown that L2 learners with a high level of self-efficacy are those who receive encouragement and assistance by the instructors (Graham et al., 2020; Xu et al., 2022), actively learn from a leading student (Wang and Sun, 2020) and willingly apply more learning strategies to enhance their involvement in class (Anam and Stracke, 2020). The study of Xu et al. (2022) showed that after an intensive summer English training program, where all four sources can be found, learners’ self-efficacy was largely improved. Also, the study of Chou (2019) on 636 high school students preparing for the college entrance examination indicated that the students’ performance in recent English tests (previous experience) and test anxiety (emotional status) can be considered two predictors of their self-efficacy toward the incoming exams. In addition, self-efficacy of learning the same second language may vary among learners with different features, for instance, the purpose of learning (Wang et al., 2018) and the learners’ first languages (Kim et al., 2021).

At the same time, self-efficacy can also influence L2 learner’s learning experience and results (Ozer and Akçayoğlu, 2021). Studies indicate that leaners with a high level of self-efficacy may feel more confident during language learning (Sabti et al., 2019), more motivated with the intention of conducting language learning activities (Mayfield and Mayfield, 2012; Doménech-Betoret et al., 2017; Anam and Stracke, 2020; Mendoza et al., 2022), and less anxious when dealing with learning issues (Yun et al., 2018; Pawlak and Csizér, 2022). Wang et al. (2021) discovered that for Chinese undergraduate students learning English, a high self-efficacy can bring them more positive emotions during the learning experience including pride and joy, and similarly, Japanese EFL learners have also felt more motivated in reading and listening when owning a positive self-efficacy (Chen et al., 2021). Also, multiple researchers have discovered that EFL learners with high level of self-efficacy tend to achieve better results in all four skills of writing, listening, reading, and speaking (Chen and Zhang, 2019; Wang and Sun, 2020; Mendoza et al., 2022).

### Relationship between foreign language anxiety and language learning motivation

Studies about the relationship between anxiety and motivation have come up with different conclusions. A large number of scholars hold the belief that anxiety and motivation are negatively correlated. For instance, Gardner et al. (1983) discovered that Canadian students who speak English may encounter different levels of anxiety in learning French, and those with better prior achievement and more motivation are likely to experience less anxious feelings. Similar results can also be found at Amiryousefi and Tavakoli’s (2011) study on test takers of TOEFL iBT and Alico’s (2016) analysis on the writing proficiency of pre-university students in the Philippines. At the same time, however, there are also some other opinions, for example, learners who are anxious may be more motivated (Strack et al., 2014) and it is motivation affects anxiety instead of the other way (Lavasani et al., 2011). Luo et al. (2020) examined Chinese college students’ attitudes and feelings in learning the second language, classified anxiety into the facilitating type and the debilitating type, and concluded that anxiety can be “significantly and positively correlated with all types of motivation” (Luo et al., 2020, p. 66). Nagle (2021) found that students’ willingness to communicate in Spanish was predicted by their attainment value and intrinsic value, and that the course achievement was predicted by their expectancy beliefs. Dong et al.’s (2022) study revealed a complex correlation between the students’ FL classroom anxiety and expectancy-value motivation: As the students’ FL classroom anxiety increased, their expectancy beliefs, intrinsic value, attainment value, and utility value decreased, but their cost value increased.

### Research questions

As discussed above, foreign language anxiety, language learning motivation, and self-efficacy are all important factors in second language acquisition and learning despite learners’ ages, nationalities, and educational backgrounds. Nevertheless, just because of the complexity of learner populations and second language learning process, more and continuous research on these issues are still called for. And although some research shows that they interact with one another and collectively affect second language learning outcomes, little research has examined their interaction simultaneously in the same situation. Moreover, there is hardly any research focusing specifically on top students’ attitudes toward learning a second language and how these attitudes affect their language proficiency. Hence, this research sought to examine English class anxiety, motivation, and self-efficacy in students from various top-notch programs of a top university in China. The following research questions were of particular interest:

1. What are the profiles of the participants in terms of English proficiency, English use anxiety, motivation, and self-efficacy?
2. What is the relationship between the participants’ English use anxiety, motivation, and self-efficacy?
3. How do English use anxiety, motivation, and self-efficacy affect the participants’ standardized test scores?

## Methodology

### Context

To foster talents and prepare them to be worldwide first-class scholars in basic yet important disciplines such as mathematics and physics, China’s Ministry of Education initiated the Top-Notch Students of Basic Disciplines Training Program in about 15 top universities in 2009. To join the program, students must do well in the National College Entrance Examinations to be admitted by a member university, and then excel in the selective tests of the Top-Notch Program of the university. Each year, the program of each university admits around 200 out of about 4,000 students it enrolls. Students in the program often enjoy more and better resources than other students of the same university. For example, they are more likely to be taught by leading professors and be granted funds for learning and research. Consequently, they generally excel in many aspects and their way of learning is expected to be enlightening to other students. Nevertheless, since 2009 when the program was first launched, not much research has been done on the program or students, even less research in second language acquisition has been done on them. This mainly motivates the present research.

The present study was conducted in a highly prestigious state-owned research-oriented university in Beijing (Quacquarelli Symonds Limited, 2021; Timers Higher Education, 2021), whose Top-Notch Program had 7 classes of varying sizes (about 15–30 students per class) every year.

### Participants

In total, 223 university students (167 male and 56 female) joined the study, and their average age is 19.67 (SD = 1.067), ranging from 16 to 22 years old. These participants are at different undergraduate years, and they are from various disciplines, including physics, mathematics, chemistry, biological science, life sciences, clinical medicine, computer science, artificial intelligence, interdisciplinary information sciences, theoretical and applied mechanics, foreign language and literature, and philosophy.

### Instruments

The participants were required to accomplish a battery of questionnaires (see Appendix) about their personal information and attitudes toward statements related to English use anxiety, motivation, and self-efficacy. The questionnaire consisted of five sections: an 11-item questionnaire of background information, an 8-item English Class Anxiety Questionnaire, a 5-item Motivational Self-Talk Questionnaire, a 3-item Self-Efficacy Questionnaire, and a 19-item Language Learning Orientations Questionnaire. Except for the first section, all statements were placed on a 5-point Likert scale with “strongly disagree” and “strongly agree” on each end.

Background Information (Item 1–11): In this section, participants’ age, gender, major, grade, and time length of English use per day were required. Also, participants’ level of English proficiency was recorded in two ways: one was the score of the most recent standardized English test they had taken, for instance, International English Language Testing System (IELTS) or College English Test (CET). The other was the self-rated proficiency of their English speaking, writing, listening, reading, and overall performance, respectively, and each item was reflected on a 1-to-10 scale, with 1 being “not at all satisfied” and 10 being “very satisfied.”

English Use Anxiety Questionnaire (Item 12–19): Gardner’s (1985) Attitude/Motivation Test Battery included an 8-item French Class Anxiety Scale that examined L2 learners’ anxious feelings of answering questions in a French class. The current study adapted this scale by changing ‘the target language’ to ‘English’ in all items, aiming to measure students’ anxiety when using English in class.

Motivational Self-Talk Questionnaire (Item 20–24): This questionnaire was adapted from that in Teng and Zhang (2016), aiming to examine how learners’ self-talk motivated themselves to continue to learn English. Since the original design was specifically for examining how learners convinced and encouraged themselves to improve their own writing skills, the current study abandoned three statements that focused on writing proficiency and rephrased the other five to fit the English learning experience in general.

Self-Efficacy Questionnaire (Item 25–27): With reference to the self-efficacy questionnaire used in Wong (2005), a three-item Self-Efficacy Questionnaire was developed in the present study: I believe that I am capable of learning English well; I believe that I know how to find the efficient way of learning English; and I believe that I can reach a high level of English proficiency someday.

Language Learning Orientations Questionnaire (Item 28–46): The questionnaire used in the current study is based on the Language Learning Orientations Scale of Noels et al. (2000), which evaluated participants’ amotivation, external regulation, introjected regulation, identified regulation, and the three aspects—knowledge, accomplishment and stimulation—of intrinsic motivation. In the current research, the amotivation section was irrelevant and thus removed, and the rest parts of the questionnaire were adapted to fit in the context of top student in top university. In general, 19 statements were listed for learners to evaluate if they had experienced motivation coming from different resources during English learning and using.

### Data collection and analysis

The questionnaires were in Chinese, and they were organized and distributed online to the participants. In total, 223 valid questionnaires were collected and analyzed via SPSS 27. Since the scores of different standard tests were provided under different marking systems (for instance the full marks are 346 for Graduate Record Examination (GRE), 120 for Test of English as a Foreign Language (TOEFL), 9 for International English Language Testing System (IELTS), and 710 for CET-4/6), they were all converted to the centesimal system for computing and analysis. Also, by averaging the scores of related items, the 35 statements of the four latter questionnaires corresponded to 9 variables for analysis: Use of English anxiety (UAE, item 12–19), Motivational Self-Talk (MST, item 20–24), Self-Efficacy in learning English (SE, item 25–27), Extrinsic Motivation – External Regulation (ER, item 28–31), Extrinsic Motivation – Introjected Regulation (IntroR, item 32–34), Extrinsic Motivation – Identified Regulation (IdenR, item 35–37), Intrinsic Motivation – Knowledge (IMK, item 38–40), Intrinsic Motivation – Accomplishment (IMA, item 41–43), and Intrinsic Motivation – Stimulation (IMS, item 44–46). Means and standard deviations of various items were firstly computed to indicate the general features of the participants, then the relations between different variables were analyzed via correlation analysis and regression analysis.

## Results

### Participants’ profiles of English proficiency, English use anxiety, motivation, and self-efficacy

According to the collected data, the participants reported using English for 2.12 h (SD = 1.10) on average per day after entering university. One hundred and ninety-one out of the 223 participants had taken part in a standardized English proficiency test like GRE, TOEFL, IELTS, CET-4, CET-6, Test for English Majors (TEM), and Tsinghua English Proficiency Test (TEPT) (English proficiency test of the university). The scores were then standardized on the scale of 1–100, which showed that the participants had an average score of 80.29 out of 100 (SD = 9.79). Data of self-ratings showed that the participants scored 4.77 to 6.50 in different aspects of English (see Table 1), indicating that the learners generally considered themselves intermediate learners of English in all aspects. Also, among all four English skills, the participants were the most satisfied with their reading proficiency (Mean = 6.50, SD = 2.03) and not so satisfied with their speaking proficiency (Mean = 4.77, SD = 2.31).

**Table 1:** Means, standard deviations and correlations of English achievements measured in different ways.

	Mean	SD	2	3	4	5	6
1. Standardized test scores	80.29	9.79	0.487^**^	0.443^**^	0.534^**^	0.443^**^	0.323^**^
2. Overall English proficiency	5.54	2.02	1	0.810^**^	0.853^**^	0.757^**^	0.793^**^
3. Speaking proficiency	4.77	2.31		1	0.821^**^	0.576^**^	0.652^**^
4. Listening proficiency	5.40	2.44			1	0.705^**^	0.676^**^
5. Reading proficiency	6.50	2.03				1	0.724^**^
6. Writing proficiency	5.22	2.07					1

** *p* ≤ 0.01.

Meanwhile, Table 1 shows that the participants’ achievements in different aspects of English were all highly positively correlated with one another, with a coefficient range of 0.323 to 0.853 (*p* ≤ 0.01). Of all the self-ratings, self-rated proficiency in overall English had the highest coefficient with standardized tests scores (*r* = 0.487) and that in speaking proficiency (*r* = 0.810), listening proficiency (*r* = 0.853), reading proficiency (*r* = 0.757), and writing proficiency (*r* = 0.793), respectively. Thus, standardized test scores and self-rated proficiency in overall English were used as indicators of students’ English achievements for further analyses in the present study.

Table 2 presents means, standard deviation, skewness, and reliability scores of the English Use Anxiety Scale (EUAS), the Motivational Self-Talk Questionnaire (MSTQ), the Self-Efficacy Questionnaire (SEQ), and the six dimensions of the Language Learning Orientations Questionnaire (LLOQ). Most of the scales were reliable in the present study, with reliability scores (Cronbach’s alpha) ranging from 0.760 to 0.894, except for the Extrinsic Motivation – External Regulation section, with a reliability of 0.489. The low reliability was that there were only four items in this scale, all adapted to fit in the context of Chinese university students, and 223 participants were not a large sample size here.

**Table 2:** Statistics of the measured variables on the scale of 1–5.

	Mean	SD	Skewness	Reliability (*α*)
English Use Anxiety Scale	2.96	0.85	0.190	0.878
Motivational self-talk questionnaire	3.56	0.87	−0.681	0.876
Self-efficacy questionnaire	3.73	0.87	−0.202	0.883
Extrinsic motivation – External regulation	2.86	0.71	0.057	0.489
Extrinsic motivation – Introjected regulation	2.14	0.90	0.562	0.822
Extrinsic motivation – Identified regulation	3.71	0.89	−0.444	0.760
Intrinsic motivation – Knowledge	3.11	1.08	0.001	0.894
Intrinsic motivation – Accomplishment	3.32	0.99	−0.187	0.875
Intrinsic motivation – Stimulation	3.70	1.06	0.240	0.863

As shown in Table 2, the participants scored 2.96 (SD = 0.85) on EUAS, 3.56 (SD = 0.87) on MSTQ, 3.73 (SD = 0.87) on SEQ, 2.86 (SD = 0.71) on external regulation, 2.14 (SD = 0.90) on introjected regulation, 3.71 (SD = 0.89) on identified regulation, 3.11 (SD = 1.08) on knowledge, 3.32 (SD = 0.99) on accomplishment, and 3.70 (SD = 1.06) on stimulation. These findings indicate that the participants reported having a low-to-medium level of anxiety when using English and a medium-to-high level of motivational self-talk and self-efficacy. Regarding the various types of motivation, they seemed to have a low level of extrinsic motivation – introjected regulation, a low-to-medium level of extrinsic motivation – external regulation and intrinsic motivation – knowledge, and a rather medium-to-high level of extrinsic motivation – identified regulation, intrinsic motivation – accomplishment and intrinsic motivation – stimulation. It also shows that all the scales had a normal distribution, with skewness values below 1.

### Relationship between the participants’ English use anxiety, motivation, and self-efficacy

Table 3 reports correlations among English use anxiety, motivation, and self-efficacy, which shows that: EUAS was significantly negatively related to SEQ (*r* = −0.441, *p* ≤ 0.01), IMK (*r* = −0.222, p ≤ 0.01) and IMS (*r* = −0.151, *p* ≤ 0.05), and it was significantly positively related to IntroR (*r* = 0.179, *p* ≤ 0.01), in which the correlation was moderate with SEQ (0.4 < |*r*| < 0.6), weak with IMK (0.2 < |*r*| < 0.4), and very weak with IMS and IntroR (0 < |*r*| < 0.2). EUAS had no significant correlations with MSTQ, ER, IdenR, and IMA. MSTQ had a significant and positive relation with SEQ (*r* = 0.325, *p* ≤ 0.01) and all six sections of LLOQ (*r* > 0, *p* ≤ 0.01), in which the correlation was weak with SEQ, ER, and IntroR (0.2 < |*r*| < 0.4) and moderate with IdenR, IMK, IMA, and IMS (0.4 < |*r*| < 0.6). SEQ had a significant and positive relation with five sections of LLOQ (*r* > 0, *p* ≤ 0.01), except the section of IntroR, which was significantly negatively related (*r* = −0.124, *p* ≤ 0.01), and the correlations were very weak (0 < |*r*| < 0.2) both ER and IntroR, weak (0.2 < |*r*| < 0.4) with both IMA and IMS, and moderate with IdenR and IMK (0.4 < |*r*| < 0.6). At last, all six sections of LLOQ (ER, IntroR, IdenR, IMK, IMA, and IMS) are significantly positively correlated with each other (*r* > 0, *p* ≤ 0.01), with the weakest correlation between IntroR and IdenR (0 < |*r*| = 0.179 < 0.2) and the strongest between IMK and IMA (0.6 < |*r*| = 0.732 < 0.8).

**Table 3:** Correlations among English use anxiety, motivation, and self-efficacy.

	1	2	3	4	5	6	7	8	9
1. EUAS	1	0.040	−0.441^**^	0.020	0.179^**^	−0.120	−0.222^**^	−0.077	−0.151^*^
2. MSTQ		1	0.325^**^	0.336^**^	0.263^**^	0.579^**^	0.526^**^	0.615^**^	0.493^**^
3. SEQ			1	0.176^**^	−0.134^*^	0.426^**^	0.443^**^	0.386^**^	0.349^**^
4. ER				1	0.463^**^	0.363^**^	0.270^**^	0.330^**^	0.374^**^
5. IntroR					1	0.179^**^	0.256^**^	0.274^**^	0.439^**^
6. IdenR						1	0.698^**^	0.681^**^	0.510^**^
7. IMK							1	0.732^**^	0.686^**^
8. IMA								1	0.691^**^
9. IMS									1

** *p* ≤ 0.01;

* *p* ≤ 0.05.

EUAS, English Use Anxiety Scale (EUAS); MSTQ, Motivational Self-Talk Questionnaire; SEQ, Self-Efficacy Questionnaire; ER, Extrinsic motivation – External Regulation; IntroR, Extrinsic motivation – Introjected Regulation; IdenR, Extrinsic motivation – Identified Regulation; IMK, Intrinsic Motivation – Knowledge; IMA, Intrinsic Motivation – Accomplishment; IMS, Intrinsic Motivation – Stimulation.

Coefficient of determination: small = *r* ≤ 0.1; medium = *r* = 0.3; large = *r* ≥ 0.5 (Cohen, 1988).

To explore whether English use anxiety, language learning motivation, and self-efficacy predicted students’ English achievements, multiple stepwise regression analyses were done, with EUAS, SEQ, and IMK being independent variables and standardized test scores and the self-rated overall English proficiency being the dependent variable, respectively. The results are reported in the tables below.

As shown in Table 4, regression analyses yielded two models with the change in R2 being 0.166 for model 1 (IMK) and 0.031 for model 2 (IMK and EUAS) for standardized test scores. Namely, IMK (intrinsic motivation-knowledge; *β* = 0.268, *t* = 3.99, *p* = 0.000, *f*2 = 0.28) and EUAS (English Use Anxiety Scale; *β* = −0.266, *t* = −3.965, *p* = 0.000, *f*2 = 0.17) were good predictors for the participants’ standardized test scores, with the first being a positive and the second a negative predictor.

**Table 4:** Multiple regression coefficients and significance of predictors for English achievements.

	*β*	*t*	*p*	VIF	Cohen’s *f*2
Model 1 with standardized test scores as the dependent variable					
IMK	0.268	3.99^**^	0.000	1.082	0.28
EUAS	−0.266	−3.965^**^	0.000	1.082	0.17
Model 2 with self-rated proficiency in overall English as the dependent variable					
EUAS	−0.465	−8.200^**^	0.000	1.242	0.07
SEQ	0.300	5.291^**^	0.000	1.242	0.17

** *p* ≤ 0.01.

Effect size of Cohen’s *f*2: small *= f*2 ≤ 0.02; medium = *f*2 = 0.15; large = *f*2 ≥ 0.35 (Cohen, 1988).

Regression analyses also yielded two models with the change in R2 being 0.357 for model 1 (EUAS) and 0.073 for model 2 (EUAS and SEQ) for self-rated proficiency in overall English. Namely, EUAS (English Use Anxiety Scale; *β* = −0.465, *t* = −8.200, *p* = 0.000, *f*2 = 0.07) and SEQ (Self-Efficacy Questionnaire; *β* = 0.300, *t* = 5.291, *p* = 0.000, *f*2 = 0.17) were good predictors for the participants’ self-rated proficiency in overall English, with the first being a negative and the second a positive predictor.

In the text here, only the significant predictors were listed. In addition, the diagnostics information indicated two outliers in each model, and despite the outliers, the histograms, normal P–P plots, and scatterplots of the model showed that most data fit the specified distribution in the models.

### Effect of English use anxiety, motivation, and self-efficacy on participants’ standardized test scores

Self-efficacy is the subjective evaluation of oneself, and it influences people’s choices, attitudes towards difficulties, performance in the process of acquisition, and the emotional status, and it thus can exert strong mediating effect on the results and output (Bandura, 1997). This section of the study examined if self-efficacy mediated the influence of English use anxiety and motivation on the participants’ English achievements, represented by the standardized test scores (STS), according to Table 5 (Baron and Kenny, 1986). In step 1, the dependent variable was the standardized test scores. With an R2 of 0.206, the standardized coefficient of EUAS was significant (*β* = −0.318, *p* = 0.000) and that of IMK was also significant (*β* = 0.246, *p* = 0.000). Then R2 in step 2 is 0.361 and the dependent variable was SE. Both standardized coefficients of EUAS (*β* = −0.405) and IMK (*β* = 0.050) were significant (*p* = 0.000). At last, all three standardized coefficients were significant when the dependent variable is the standardized test score. The data for each term were SE (*β* = 0.185, *p* = 0.022), EUAS (*β* = −0.243, *p* = 0.001) and IMK (*β* = 0.183, *p* = 0.012). Since the effects of both EUAS and IMK decreased after SE was introduced into the model, the influences of both EUAS and IMK on STS were partially mediated by SE (Wen et al., 2004).

**Table 5:** Multiple regression analysis for examining the mediating effect.

	Unstandardized coefficients		Standardized coefficients	*t*	*p*	VIF	*R* square	Adj. *R* square	*F*
	*B*	Std. error	Beta						
Step 1^a^^,^^c^									
Constant	85.08	3.463		24.565	0.000^**^		0.206	0.198	*F* (2188) = 24.449, *p* = 0.000
EUAS	−3.699	0.789	−0.318	−4.69	0.000^**^	1.088			
IMK	2.236	0.615	0.246	3.636	0.000^**^	1.088			
Step 2^b^^,^^d^									
Constant	4.092	0.281		14.567	0.000^**^		0.361	0.354	*F* (2188) = 53.080, *p* = 0.000
EUAS	−0.426	0.064	−0.405	−6.664	0.000^**^	1.088			
IMK	0.281	0.05	0.343	5.635	0.000^**^	1.088			
Step 3^a^^,^^c^									
Constant	79.694	4.996		15.35	0.000^**^		0.228	0.216	*F* (3187) = 18.445, *p* = 0.000
SE	2.05	0.889	0.185	2.305	0.022^*^	1.565			
EUAS	−2.826	0.867	−0.243	−3.259	0.001^**^	1.345			
IMK	1.66	0.657	0.183	2.526	0.012^*^	1.272			

** *p* ≤ 0.01;

* *p* ≤ 0.05.

a Dependent Variable: STS.

b Dependent Variable: SE.

c D-W: 1.999.

d D-W: 1.888.

## Discussion

### Participants’ profiles of English proficiency, English use anxiety, motivation, and self-efficacy

Firstly, when dealing with English use and learning, the participants experienced a medium-to-low level of anxiety, and the fact that English use anxiety and the participants’ language proficiency (reflected both in standardized language test scores and self-rated scores) were significantly negatively correlated was in accordance with the discoveries of multiple previous researchers (Gardner and Mac Intyre, 1993; MacIntyre et al., 1998; Onwuegbuzie et al., 1999; Elkhafaifi, 2005). Secondly, they had a medium-to-high level of self-efficacy and conducted motivational self-talks quite often, which may explain the difference between participants’ standardized test scores and their self-evaluation of English proficiency. Despite the fact that the average score in language tests was as high as 80.29%, their self-evaluation was only at a medium stage (5.54 out of 10), and the separate scores for the four English skills were 6.50 for reading, 5.40 for listening, 5.22 for writing, and 4.77 for speaking. The participants tended to give a rather modest evaluation on their English proficiency, this action fit with their medium-to-high level of self-efficacy (Bandura, 1986), which then led to setting the goal lower than their actual abilities and gain more satisfaction when they actually reached a high level of result. Therefore, the students could be highly motivated by identified regulation, which is the type of extrinsic motivation with the highest level of self-determination (Noels et al., 2000), and they were also intrinsically motivated by sensation of mastering the skill (IM – Accomplishment) and excitement of fulfilling the work (IM – Stimulation). Meanwhile, the students only had a low or low-to-medium level of the other two types of extrinsic motivation (introjected and external regulation), and the intrinsic motivation of acquiring knowledge was also at a low-to-medium level. For these top students majoring in different subjects, learning English was no longer a compulsory requirement of the university, so they were more motivated by their internal satisfaction and personal needs but less influenced by external factors.

The regression analyses of how anxiety and motivation affect English achievements were conducted, and the results indicated a significant influence on both the scores of standardized tests and the self-rated proficiency. The effect of learning motivation was positive, and students who were more motivated tended to perform better in language tests and consider themselves more proficient in using English. Oppositely, the impact of English use anxiety could be adverse, interfering the learners’ performance in taking language tests and causing them to underestimate their own language proficiency.

### Relationship between the participants’ English use anxiety, motivation, and self-efficacy

English use anxiety and language learning motivation were negatively correlated in the current study, and the correlation was specifically significant between anxiety and intrinsic motivation – knowledge, as well as between anxiety and intrinsic motivation – stimulation. Referring to the statements of the questionnaires, English L2 learners who are more anxious with using the language may be feel less willing to joining the course or related activities, which then influences the process of knowledge acquisition. In this way, the intrinsic motivation towards learning new information will be diminished. Also, without enough knowledge learned, the capability of speaking (MacIntyre, 2007), writing (Alico, 2016), reading (Saito et al., 1999), and listening (Amiryousefi and Tavakoli, 2011) may all be affected, and the feeling of success or accomplishment can hard be realized. Therefore, the motivation of stimulation cannot be triggered. In all, the discoveries of the current study are consistent with that of many previous researchers on the relationship between English use anxiety and learning motivation (Gardner et al., 1983; Amiryousefi and Tavakoli, 2011; Alico, 2016). Also, the results of the analysis indicated that learners with high self-efficacy tend to be less anxious, more confident and more motivated in language learning, which is consistent with the previous studies of how self-efficacy influences language learning emotions and attitudes (Mayfield and Mayfield, 2012; Anam and Stracke, 2020; Wang et al., 2021; Mendoza et al., 2022; Pawlak and Csizér, 2022).

### Effect of English use anxiety, motivation, and self-efficacy on participants’ standardized test scores

The mediating effect of self-efficacy was also proved in the study. Firstly, a high level of self-efficacy emphasizes on the learners’ self-confidence in accomplishing the learning assignments and language tests, so that the learners are more determined in their language skills, which can balance the negative effect caused by the anxiety (Wong, 2005) and lead to a better result in the standardized tests. Also, instead of being fully motivated by the intrinsic desire toward new knowledge, these participants from universities, who may feel less necessary to acquire new knowledge in language learning, can be motivated by their own self-efficacy in the general experience of language learning, which offers them more confidence in handling the language skills they have already owned and applying the skills to language tests for higher scores. In other words, the participants’ self-efficacy may affect the original influence of either anxiety of motivation by reducing the negative attitude and providing one more source of positive feeling, which then helps them to reach better language achievements than those with low self-efficacy (Mayfield and Mayfield, 2012; Sabti et al., 2019).

## Conclusion and implications

The present study explored how English use anxiety, language learning motivation, and self-efficacy were related to one another and how they collaboratively predicted English achievements of students in top-notch programs of a top university in China. Major findings were:

1. The participants had a low-medium level of English use anxiety, and among different types of motivations, extrinsic motivation – introjected regulation was the least detected (low level), extrinsic motivation – external motivation and intrinsic motivation – knowledge was the next in line (low-to-medium level), while extrinsic motivation – identified regulation, intrinsic motivation – accomplishment and intrinsic motivation – stimulation were mostly recognized (medium-to-high level). Also, the participants had a medium-to-high level of motivational self-talk and self-efficacy.
2. English use anxiety and language learning motivation were negatively correlated with each other, and to be more specific, English use anxiety was significantly negatively correlated with both intrinsic motivation – knowledge and intrinsic motivation – stimulation.
3. English use anxiety significantly negatively predicted the learners’ English achievements, while intrinsic motivation-knowledge and self-efficacy significantly positively predicted the learners’ standardized test results and self-rated proficiency in overall English, respectively.
4. Self-efficacy of the learners mediated the influence of anxiety and motivation on the top students’ English achievements.

These findings further pinpoint the importance of English use anxiety, motivation, and self-efficacy in second language acquisition and learning. They also brought attention to the affect of top students of top universities, focusing on how these leading learners of various disciplines evaluate their language use anxiety, motivation, and self-efficacy. The results showed that for top students in various academic fields, learning and using English still occupies a part of their daily life, and they are mostly motivated by internal determination of accomplishing English-related tasks and the excitement of reaching the goals instead of the external regulations. Consistent with previous research, participants in current studies also experienced negative effects of anxiety and positive influence of motivation on their language achievements, and those with high self-efficacy can deal with the effect better. Besides conveying academic information, L2 teachers should pay more attention to understanding and sensing learners’ attitudes and emotions, and build up efficient regulatory strategies to help the learners cope with tension and stay motivated, so that to reach better performance in both teaching and learning. At the same time, L2 learners, especially the self-taught ones, should be aware of the emotional changes and take active steps to keep a positive attitude toward the learning experience.

The current study has some certain limitations. Firstly, the participants took part in different types of standardized tests, and these tests do not always share the same evaluation criteria or level of difficulty. Though the self-evaluation of the participants was also included to provide a more comprehensive image of learner’s language proficiency in the current study, future studies could still discover better ways to describe the participants’ language skills in a more unified manner. Also, this research did not continue to discover how L2 learners cope with their anxiety or develop their motivation, which can be examined by further studies of learning strategies and learning styles. Another aspect that can be included in the following studies is the individual differences among the participants, for instance how English learning has benefited their university life and study of their own majors. These qualitative studies could be realized via interviews or open-ended questionnaires.

## Data availability statement

The raw data supporting the conclusions of this article will be made available by the authors, without undue reservation.

## Author contributions

XW: design of study, analysis and interpretation of data, writing-review and editing, and supervision. HY: writing-original draft and analysis and interpretation of data. JL and ZL: data collection. All authors contributed to the article and approved the submitted version.

## Funding

This research was sponsored by the 2021 Top-Notch Students of Basic Disciplines Training Program 2.0 Project (No. 20211008).

## Conflict of interest

The authors declare that the research was conducted in the absence of any commercial or financial relationships that could be construed as a potential conflict of interest.

## Publisher’s note

All claims expressed in this article are solely those of the authors and do not necessarily represent those of their affiliated organizations, or those of the publisher, the editors and the reviewers. Any product that may be evaluated in this article, or claim that may be made by its manufacturer, is not guaranteed or endorsed by the publisher.

## Supplementary material

The Supplementary material for this article can be found online at: https://www.frontiersin.org/articles/10.3389/fpsyg.2022.953600/full#supplementary-material
