# Supplementary material for: English use anxiety, motivation, self-efficacy, and their predictive effects on Chinese top university students’ English achievements
Source: Front Psychol. 2022 Oct 19;13:953600. doi: 10.3389/fpsyg.2022.953600 (PMC9626819; doi:10.3389/fpsyg.2022.953600)
Supplement: Supplementary file 1 [file Data_Sheet_1.pdf]

APPENDIX:

Questionnaires on Top Students' English Use and Learning

Section One:

1. Age:_________________
2. Gender: _________________
3. Major/Program: _________________
4. Year of study: _________________
5. I have been using English for _____ hours per day after entering university.
   a. ≦1 hour b. 1.5-2.5 hours c. 3-4.5 hours d. ≧5 hours
6. The most recent standardized English test that I have taken was ____________________, and the score I got was ____________________.

Please choose the option that fits your condition the most. (1 = not at all satisfied, 10 = very satisfied)

7. My evaluation on my overall English proficiency: ( )
8. My evaluation on my speaking proficiency in English: ( )
9. My evaluation on my listening proficiency in English: ( )
10. My evaluation on my reading proficiency in English: ( )
11. My evaluation on my writing proficiency in English: ( )

Section Two:

Please choose the option that fits your condition the most.
(1= strongly disagree, 2 = disagree, 3 = not sure, 4 = agree, 5 = strongly agree)

12. I don't usually get anxious when I have to respond to a question in English. ( )
13. I am always afraid that the other students would laugh at me if I speak up in English. ( )
14. I always feel that the other students are more at ease than I am in English learning. ( )
15. I am never embarrassed to volunteer answers in English. ( )
16. I am generally tense whenever participating in activities in English. ( )
17. I never understand why other students are so nervous when using English. ( )
18. I usually feel relaxed and confident when participating in activities in English. ( )
19. Whenever I have to answer a question in English, out loud, I would get nervous and confused. ( )
20. I tell myself that I need to keep studying to improve my English competence. ( )
21. I persuade myself to keep on learning English to find out how much I can learn. ( )
22. I tell myself that I should keep on learning English to become good at it. ( )
23. I remind myself about how important it is to get good grades in English. ( )
24. I tell myself that it is important to practice English to outperform my peers. ( )
25. I believe I have the ability to learn a language successfully. ( )
26. I believe I have the ability to get the score I am trying for in my next English test. ( )
27. I believe I know how to find an effective way to learn English. ( )
28. I learn English because it is a required class. ( )
29. I learn English because I want to have high GPA. ( )
30. I learn English because I want to study abroad later. ( )
31. I learn English because I want to travel in English-speaking countries. ( )
32. I learn English because I want to prove I am a good student. ( )
33. I learn English because if I do poorly in it, I will feel I lose face. ( )
34. I learn English because my friends all learn English well. ( )

35. I learn English because it is important to my future development. (   )
36. I learn English because I want to master a foreign language. (   )
37. I learn English because I want to be someone who is good at English. (   )
38. I learn English for the pleasure I experience in learning. (   )
39. I learn English for the good feeling I get in learning it well. (   )
40. I learn English for the satisfied feeling I get in knowing new things of English. (   )
41. I learn English for the pleasure I experience when I do well in English. (   )
42. I learn English for the enjoyment I experience when having a high score in exams. (   )
43. I learn English for the satisfied feeling when I master difficult words. (   )
44. I learn English for the good feeling when I hear English spoken by other people. (   )
45. I learn English for the good feeling when I speak English. (   )
46. I learn English for the pleasure I get from knowing a difficult English words around me. (   )
